# Macrófagos e Neovascularização na Neoaterosclerose Intra-Stent: um Fenótipo Inflamatório Acelerado por OCT com Implicações Terapêuticas

**DOI:** 10.36660/abc.20220732

**Published:** 2022-11-23

**Affiliations:** 1 Universidade Federal de São Paulo Escola Paulista de Medicina São Paulo SP Brasil Escola Paulista de Medicina, Universidade Federal de São Paulo, São Paulo, SP – Brasil

**Keywords:** Reestenose Coronária, Fenótipo/inflamação, Intervenção Coronária Percutânea, Aterosclerose, Diagnóstico por Imagem/métodos

Nesta edição dos Arquivos Brasileiros de Cardiologia, Pinheiro et al.,^1^ apresentam dados de tomografia de coerência óptica (OCT) com diferenças claras de inflamação e neovascularização entre aterosclerose de novo, reestenose intra-stent por hiperplasia intimal e neoaterosclerose intra-stent.

Pacientes submetidos à intervenção coronária percutânea com implante de stent podem apresentar sintomas recorrentes de doença coronariana devido à reestenose intra-stent por lesão vascular que desencadeia uma resposta proliferativa na íntima,^2^ minimizada pelas técnicas atuais e stents de nova geração.^3^ No entanto, a lesão neointimal secundária a um atraso na neoendotelização pode levar à recorrência dos sintomas, geralmente durante o primeiro ano de intervenção coronariana.^4^

Em pacientes com síndromes coronarianas agudas, respostas inflamatórias pronunciadas podem ser detectadas por semanas,^5^ contribuindo para a instabilidade da placa^6^ e massa infartada e remodelação ventricular após infarto do miocárdio.^7^ Além disso, o implante de stent também promove inflamação sistêmica e local.^8^

Russel Ross^9^ definiu a aterosclerose como uma doença inflamatória.^9^ Pinheiro et al.,^1^ relataram aumento da atividade inflamatória e neovascularização entre lesões de novo e neoaterosclerose intra-stent. Essas duas formas de aterosclerose podem ter diferenças importantes na fisiopatologia. A aterosclerose em artérias nativas está relacionada a fatores de risco cardiovascular e leva muito tempo para se desenvolver, mas após síndromes coronarianas agudas e/ou implante de stents, a inflamação sistêmica pode acelerar sua progressão,^6^ A neoaterosclerose intra-stent é uma forma nova e rápida de aterosclerose relacionada à lesão e inflamação vascular.^4^

Complicações como a ruptura da placa aterosclerótica parecem estar associadas não apenas à expansão da placa,^10^ mas também a características de maior vulnerabilidade (conteúdo inflamatório, capa fibrosa fina e maior teor lipídico).^11^ A OCT abordou adequadamente todos esses aspectos. As descobertas do artigo têm implicações importantes:

1) A necessidade de terapia apropriada (incluindo terapias hipolipemiantes altamente eficazes) para prevenir o desenvolvimento de aterosclerose em artérias coronárias nativas e possivelmente na aterosclerose intra-stent.^12,13^
2) O estudo levanta o debate sobre a relevância do risco inflamatório residual e a oportunidade do uso de anti-inflamatórios.^14,15^
3) Mais estudos são necessários para entender melhor a neoaterosclerose intra-stent e a necessidade de estratégias mais abrangentes, para prevenir esta forma de falha do stent.
