# Supplementary material for: Macrófagos e Neovascularização na Neoaterosclerose Intra-Stent: um Fenótipo Inflamatório Acelerado por OCT com Implicações Terapêuticas
Source: Arq Bras Cardiol. 2022 Nov 23;119(6):938–9. [Article in Portuguese] doi: 10.36660/abc.20220732 (PMC9814806; doi:10.36660/abc.20220732)
Supplement: Translation

# Translation: Macrophages and Neovascularization in In-Stent Neoatherosclerosis: An Accelerated Inflammatory Phenotype by OCT with Therapeutic Implications

**Affiliations:** 1 São Paulo SP Brazil Escola Paulista de Medicina, Universidade Federal de São Paulo, São Paulo, SP – Brazil

**Keywords:** Coronary Restenosis, Phenotype/inflammation, Percutaneous Coronary Intervention, Atherosclerosis, Diagnostic, Imaging/methods

In this edition of *Arquivos Brasileiros de Cardiologia*, Pinheiro et al.^1^ show optical coherence tomography (OCT) data with clear differences in inflammation and neovascularization among de novo atherosclerosis, in-stent restenosis due to intimal hyperplasia, and in-stent neoatherosclerosis.

Patients undergoing percutaneous coronary intervention with stent implantation may have recurrent symptoms of coronary heart disease due to in-stent restenosis due to vascular injury that triggers an intimal proliferative response,^2^ minimized by current techniques and stents of new generation.^3^ However, the neointimal lesion secondary to a delay in neoendothelialization can lead to symptom recurrence, usually during the first year of coronary intervention.^4^

In patients with acute coronary syndromes, pronounced inflammatory responses can be detected for weeks,^5^ contributing to plaque instability^6^ and infarcted mass and ventricular remodeling after myocardial infarction.^7^ In addition, stent implantation also promotes systemic and local inflammation.^8^

Russel Ross^9^ defined atherosclerosis as an inflammatory disease.^9^ Pinheiro et al.^1^ reported increased inflammatory activity and neovascularization among de novo lesions and intra-stent neoatherosclerosis. These two forms of atherosclerosis may have important differences in pathophysiology. Atherosclerosis in native arteries is related to cardiovascular risk factors, and it takes a long time to develop, but after acute coronary syndromes and/or stent implantation, systemic inflammation may accelerate its progression.^6^ Intra-stent neoatherosclerosis is a new and fast form of atherosclerosis-related to vascular injury and inflammation.^4^

Complications such as atherosclerotic plaque rupture seem to be associated not only with plaque expansion^10^ but also with characteristics of greater vulnerability (inflammatory content, thin fibrous cap, and higher lipid content).^11^ OCT has properly addressed all these aspects. The article’s findings have important implications:

The need for appropriate therapy (including highly-effective lipid-lowering therapies) to prevent the development of atherosclerosis in native coronary arteries and possibly in the intra-stent atherosclerosis.^12,13^

The study raises the debate about the relevance of residual inflammatory risk and the opportunity to use anti-inflammatory drugs.^14,15^

More studies are necessary to understand better intra-stent neoatherosclerosis and the need for more comprehensive strategies, to prevent this form of stent failure.
